# *Bacillus safensis* LAU 13: a new source of keratinase and its multi-functional biocatalytic applications

**DOI:** 10.1080/13102818.2014.986360

**Published:** 2014-12-01

**Authors:** Agbaje Lateef, Isiaka Adedayo Adelere, Evariste Bosco Gueguim-Kana

**Affiliations:** ^a^ Microbiology Unit, Department of Pure and Applied Biology, Faculty of Pure and Applied Sciences, Ladoke Akintola University of Technology, Ogbomoso, Nigeria; ^b^ Department of Microbiology, Faculty of Science, Federal University of Technology, Minna, Nigeria; ^c^ Department of Microbiology, School of Life Science, University of KwaZulu-Natal, Pietermaritzburg, South Africa

**Keywords:** *Bacillus safensis*, keratinase, feather, dehairing, destaining, biocatalysis

## Abstract

A newly isolated bacterium identified as *Bacillus safensis* based on biochemical tests and 16S rRNA analysis and its mutant variant created by exposure to ultraviolet radiation at 254 nm were investigated for keratinolytic activity. The wild-type strain produced 35.4–50.4 U/mL keratinase over a period of 120 h, while the mutant one yielded 64.4–108.5 U/mL keratinase for the same period of 120 h. The optimal conditions for the enzyme activities were pH 7.5 and 40 °C. The mutant and wild-type strain keratinases retained 59% and 54% of their activity after 12 h pretreatment at 40 °C, and 64% and 60% of their activity after 12 h at pH 7.5, respectively. The keratinases showed high substrate specificity for feathers, but low specificity for human and bovine hairs. The enzymes were activated by Na^+^, Ca^2+^, Fe^2+^ and Mg^2+^. However, while Mn^2+^ activated the enzyme from the mutant strain, it inhibited that of the wild type. The mutant and wild-type strain completely degraded whole chicken feathers after 6 and 9 days at 30 ± 2 °C, and also completely dehaired goat skin within 12 and 16 h, respectively, without damage to the skin. Similarly, remarkable destaining of blood-stained cloth occurred within 2–3 h. The obtained results showed an improvement in the properties of the mutant strain for use of the micro-organism or its enzyme as biocatalysts.

## Introduction

Keratins are insoluble fibrous proteins found in hair, wool, feather, nail, horns and other epithelial covering, with rich beta helical coils that are linked through cysteine bridges.[1–3] These keratinous substrates are considered as one of the major factors, which contribute to pollution, due to their recalcitrant nature,[4] as a result of the strong structural stabilization by several hydrogen and disulphide bonds, cysteine bridges and hydrophobic interactions.[5] The development of enzymatic and/or microbiological methods for the hydrolysis of feathers into soluble proteins and amino acids could be extremely attractive as a cheap and easy method for production of valuable products.

Keratinases (E.C 3.4.21/24/99.11) are a class of proteases capable of cleaving different keratin-containing substrates.[4] They are mainly serine or metalloproteases that possess the ability to degrade the structures that form keratinous proteins.[6] Keratinolytic activity has been reported for various bacterial genera,[7–11] fungal species [12,13] and actinomycetes [14], with the enzyme produced both in submerged and solid state fermentations. There are also reports of improvement of keratinolytic activities of bacterial strains through mutagenesis by exposure to ultraviolet (UV) light.[15,16]

Keratinolytic enzymes are novel biocatalysts that are applicable in detergent, medical, cosmetic, textile and leather industries, in the degradation of prion, as pesticides, production of biodegradable films, glues and foils.[12,17,18] Therefore, efforts are continuously intensified to screen the environment for the isolation of novel keratinase-producing strains.

In this study, we report the isolation of a novel strain of *Bacillus safensis* for the production of keratinase and induction of its mutant variant, using UV radiation. The produced keratinases demonstrated remarkable feather-degrading, dehairing and destaining abilities, indicating the potential of the isolate in diverse biotechnological processes to create novel bio-products.

## Materials and methods

### Preparation of substrates and media

Feathers and soil were obtained from the feather dump site of LAUTECH Teaching and Research Farm, Ogbomoso, Nigeria. Feathers were processed to 60-mesh particle sized powders, as previously described.[7] The powders were kept at room temperature (30 ± 2 °C) and used for further studies. The minimal medium was prepared as follows: 2 g/L of NaNO_3_; 2 g/L of NaCl; 2 g/L of KH_2_PO_4_; 0.05 g/L of MgSO_4_; 0.1 g/L of FeSO_4_·7H_2_O; 0.1 g/L of CaCO_3_; 20 g/L of keratin substrate and 20 g/L of agar-agar. The medium was sterilized at 121 °C for 15 min, and then supplemented with 0.05 g/L of sterile nystatin to inhibit the growth of fungi.[7]

### Isolation of micro-organisms

About 1 g of soil sample was serially diluted using distilled water. Inoculation was done by using 0.2 mL of the 10-fold dilution aliquot on the minimal medium for the selective growth of isolates, using the pour plate method. The plates were incubated at 37 °C for up to 72 h. Distinct colonies with characteristic morphological features were selected, isolated and purified on yeast extract agar (Lab M Ltd, UK) to obtain pure cultures. The pure cultures were stored on agar slants of yeast extract agar and minimal selective medium at 4 °C until needed.

### Biochemical and molecular characterization of the isolates

The biochemical tests, viz. Gram-staining, motility, indole production, methyl red, Voges Proskauer's, citrate utilization, sugar utilization, spore staining, catalase, oxidase, coagulase, urease, hydrogen sulphide and hydrolysis of starch were carried out according to the methods of Brenner et al.[19] Genomic DNA was isolated from single colonies of the *Bacillus* isolate, as described by Sambrook et al.[20] The DNA fragments were amplified using polymerase chain reaction at 68–72 °C with BacF (5′-GGGAAACCGGGGCTAATACCGGAT-3′) and R1378 (5′-CGGTGTGTACAAGGCCCGGGAACG-3′) as the forward and reverse primer, respectively. The amplification was carried out using 1 U Taq DNA polymerase (Bio-Lab Ltd., Auckland, New Zealand) in a G-STORM Thermal cycler (Vacutec, South Africa) under the following conditions: 95 °C for 5 min, followed by 25 cycles of 95 °C for 1 min, 50 °C for 30 s and 72 °C for 1.5 min, and finally 72 °C for 5 min. The DNA fingerprints were separated electrophoretically on 1.0% agarose gel with tris-acetate-EDTA (TAE) as running buffer. The gel was stained with 6X loading dye (Thermo Scientific, USA). Two replicates of the 16S rRNA gene (approximately 1500 bp) were sequenced using Sanger's method. These sequences were browsed in the database of the National Centre for Biotechnological Information (NCBI) (http://blast.ncbi.nlm.nih.gov/) via the blastn option for possible matches and were thereafter submitted to GenBank (www.ncbi.nlm.nih.gov/genbank) under accession number KJ461434.

### Mutagenesis

Mutagenesis was carried out by the modified method of Evans et al.[21] Bacterial cultures were streaked on the entire surface of the feather agar plates. Plates (in triplicates), with the lids removed, were exposed to UV light of 254 nm at a distance of 30 cm for 5, 10, 15 and 20 min. After the exposure, the plates were incubated at 37 °C for 48 h. The largest distinct colonies that developed were selected for further studies.

### Inoculum development

A loopful of pure culture was inoculated into an inoculum medium consisting of 1% feather meal and 0.2% yeast extract (pH 7.5). The culture was incubated at 37 °C and 100 r/min for 24 h.

### Production of keratinase

Production of keratinase was carried out by inoculating 1 mL of inoculum into 19 mL of fermentation medium (see Preparation of substrates and media, but without agar and nystatin) in 100 mL flasks. The flasks were incubated at 37 °C at 100 r/min for up to 120 h. At 24 h interval, whole flasks were taken out, the broth was centrifuged at 5000 r/min at 10 °C for 20 min and the supernatants served as crude extracellular keratinases, which were used without further purification. When not used immediately, the crude enzymes were stored at 4 °C. The pH values of culture broths and the keratinolytic activities of supernatants were determined. All readings were taken in triplicates.

### Determination of keratinolytic activity

Keratinase activity was determined by the modified method of Cheng et al.[22] The reaction mixture consisted of 0.5 mL of crude enzyme, 1.5 g of feather powder and 2 mL of phosphate buffer (pH 7.5). The control experiment was made up of buffer and feather powder only. The reaction was carried out at 40 °C for 3 h with shaking at 100 r/min. Subsequently, the reaction was stopped by adding 2 mL of 10% trichloroacetic acid. Precipitated proteins were removed by centrifugation at 5000 r/min for 15 min. The increase in absorbance at 280 nm of the filtrate of the test sample relative to that of the control was taken as a measure of release of protein. This was converted into keratinase units (1 U = 0.01 absorbance increase for 1 h reaction time).[23]

### Effects of pH and temperature on keratinolytic activity

The effect of pH was studied by assaying the enzyme activity, using citrate buffer with pH 5.0 or 5.5, phosphate buffer with pH 6.0, 6.5, 7.0, 7.5, 8.0, 8.5 and bicarbonate buffer with pH 9.0, 10.0 and 11.0. The effect of temperature was measured by incubating the enzyme at temperatures ranging from 30 to 60 °C at the optimum pH. At the end of the reaction, the residual activity of the enzyme was determined.[7]

### pH and temperature stability of keratinase

The enzyme was incubated together with an equal amount of phosphate buffer (pH 7.5) at room temperature (30 ± 2 °C) for 12 h. The enzyme activity was determined against a control experiment without pH pretreatment. The thermal stability of the enzyme was determined by incubating the enzyme alone in a water bath at 40 °C for 12 h. The residual enzyme activity was determined against a control experiment without thermal pretreatment.

### Substrate specificity of crude keratinase

The effect of different substrates on keratinase activity was estimated using feather, bovine and human hair.

### Effects of metal salts on keratinase activity

The enzyme activity was determined in the presence of 10 mmol/L of NaCl, CaCO_3_, ZnSO_4_, MnCl_2_, HgCl_2_, CuSO_4_, FeSO_4_ and MgSO_4_. The enzyme activity was determined against a control experiment containing only keratinase, feather powder and phosphate buffer (pH 7.50) to obtain the residual activity.

### *In situ* degradation of whole intact feather

The ability of both the wild-type and the mutant strain to degrade a native keratin substrate was investigated by suspending a whole chicken feather in minimal medium (pH 6.5), followed by sterilization at 121 °C for 15 min. This was then inoculated with 5% (v/v) of inoculum. The tubes were incubated at 100 r/min and room temperature (30 ± 2 °C) for several days. The control experiments lacked inoculum. The tubes were visually examined on a daily basis to determine the level of degradation of the whole feather.[7]

### Dehairing of goat hide

Fresh goat skin was obtained from a local butcher and incubated in a solution of chloroform and 90% ethanol (2:1) for 2 h in order to remove lipids and fats.[24] The skin was then washed with detergent and water to remove impurities and dried in an oven at 60 °C overnight. The skin was kept at 4 °C until further use. Goat skin pieces (2 cm^2^ × 4 cm^2^) were incubated in 50 mL of crude keratinase at room temperature (30 ± 2 °C). At intervals of 4 h, skins were visually inspected for dehairing. Chemical dehairing was performed with 10% lime and 2% sodium sulphide for 24 h.

### Destaining of blood-stained fabric

Wash performance of the crude keratinases from both the mutant and the wild-type strain was evaluated by using them on blood stains on white cotton fabrics, using a modification of the method of Kumar and Bhalla.[25] Clean white cotton test fabric pieces (4 cm^2^ × 4 cm^2^) were stained with chicken blood. The stained pieces were allowed to dry. They were then added into 250 mL Erlenmeyer flasks containing 57.4 and 89.2 U/mL of crude enzyme from the wild-type and the mutant strain in 100 mL reaction mixture, respectively. The flasks were incubated at room temperature (30 ± 2 °C). A control experiment was conducted under similar conditions, except that no enzyme was added. Stain removal was visually monitored by washing the clothes with tap water.

## Results and discussion

### Bacterial isolates

Four bacterial strains were isolated from the screened feather dumping site, amongst which *Bacillus safensis* LAU 13 (GenBank accession no. KJ461434) showed highest keratinolytic activity (KA). This strain is a rod-shaped, Gram-positive and spore-forming bacterium. The analysis based on 16S rRNA sequence showed that its sequence has 100% sequence homology with *B. safensis* CBN-8 (JQ353775). Degradation of keratin has been reported to be mostly confined to Gram-positive bacteria, including *Bacillus*, *Lysobacter* and a few strains of Gram-negative bacteria, viz. *Vibrio* and *Xanthomonas*.[26] There are previous reports on the isolation of keratinase-producing strains of *B*. *subtilis*, *B*. *licheniformis*, *B*. *pumilus*, *B*. *cereus*, *B*. *halodurans* and *B*. *weihenstephanensis*,[7,8,27–36] but, to the best of our knowledge, there is no such report on *B*. *safensis*. Therefore, this study represents the first reference to *B. safensis* as a producer of keratinase.

*B*. *safensis* was first identified in 2006 as a contaminant from spacecraft–assembly facilities in USA from which it derived its specific epithet ‘safensis’.[37] The isolate has been reported as an endophytic,[38], salt-loving plant growth promoting rhizobacterium.[39,40] These bacteria have also been reported to contaminate fresco surfaces,[41] soil,[42] chilli and eggplant,[43] *Populus euphratica*,[44] marine zooplanktons,[45] *Tilapia* [46] and termite gut [47] in countries, such as the USA, India, China, Brazil, Egypt and Turkey.

The production of industrially important enzymes, such as β-galactosidase,[48] endo-inulinase [49] and lipases [50] by some strains of *B. safensis* has been reported. In addition, Berrada et al. [51] used *B. safensis* CCMM B582 for biological control of tomato grey mould caused by *Botrytis cinerea*. Therefore, the production of keratinase by an indigenous strain of *B. safensis* LAU 13 adds to the increasing industrial potential of this bacterium. We have also shown the potential of the crude keratinase of *B. safensis* LAU 13 in the green synthesis of silver nanoparticles.[52]

### Kinetics of keratinolytic activity and pH change during the growth of *B. safensis* LAU 13

**Figure 1.**
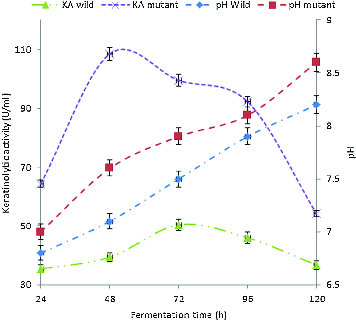
Kinetics of KA (KA) and pH change during the growth of *B. safensis* LAU 13 wild-type and mutant strain.

The keratinolytic activity (KA) of both the wild-type and the mutant strain grown on feather substrate were different, as shown in Figure 1. The KA in the crude extracellular extract from the wild-type isolate was in the range of 35.4–50.4 U/mL during 120 h of cultivation using feathers as a keratin substrate. The enzyme activity reached a maximum of 50.4 U/mL at 72 h of cultivation. Thereafter, the titre of the enzyme decreased to reach a final value of 36.7 U/mL at 120 h of cultivation. However, the KA of the mutant strain enzyme was in the range of 64.4–108.5 U/mL during the same period of cultivation. The maximum enzyme activity was 108.5 U/mL at 48 h of cultivation. The KA of the mutant strain was about 2.2-fold higher than that of the wild-type strain. Several authors have reported similar keratinolytic activities for some bacilli. For instance, Lateef et al. [7] previously reported KA of 51.7 U/mL for *Bacillus cereus* LAU 08 isolated from Nigerian soil when cultivated on feather substrate, while Son et al. [53] reported KA in the range of 14–16.7 U/mg for *Bacillus pumilus* with the same substrate. Also, keratinolytic *Bacillus* sp. AJ4 and *Bacillus* sp. AJ9 isolated from feather dumped soil showed KA of 82 and 78 U/mL as reported by Arasu et al.[54] The improvement in the KA of the mutant strain in our study may be attributed to the mutagenic effect of UV light. Wu et al. [16] reported a UV-light induced mutant strain of *Stenotrophomonas maltophilia* DHHJ with KA 1.3-fold higher than that of its wild-type strain.

The kinetics of pH change during the growth of the wild-type and the mutant strain in feather medium is also shown in Figure 1. During the growth of the wild-type isolate, the pH of the culture medium increased to the highest value of 8.2 (from an initial value of 6.0) at 120 h of cultivation, while the highest value of 8.6 was obtained for the mutant strain, thus indicating a drift towards alkalinity. It has been previously reported that alkalinity usually accompanies keratinolysis,[26,55] as observed in this study.

### Effect of pH and temperature on keratinolytic activity

**Figure 2.**
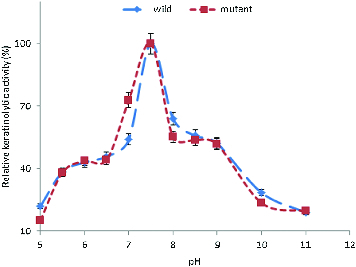
Effect of pH on the KA of *B. safensis* LAU 13 wild-type and mutant strain.

Induced by feathers, the crude extracellular keratinases of the wild-type and the mutant strain were active over a broad range of pH (5–11), with more than 50% activity occurring in the pH range between 7.0 and 9.0. The KA increased from pH 5.0 and reached its maximum at pH 7.5 (Figure 2). It has been reported that keratinases from most bacteria, actinomycetes and fungi have a pH optimum in the neutral to alkaline range.[28,56–64]

**Figure 3.**
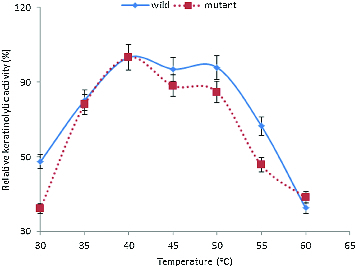
Effect of temperature on the KA of *B. safensis* LAU 13 wild-type and mutant strain.

The enzymes were also active within the temperature range of 30–60 °C (Figure 3). The KA increased at 30 °C to reach an optimum at 40 °C. However, more than 80% activity was obtained between 35 and 50 °C. The optimum temperatures of keratinases have been reported to range from 30 to 80 °C.[7,9,11,26] These properties indicated that this enzyme could be a very relevant tool in various biotechnological processes.

### pH and temperature stability of keratinases

The results from the thermostability assay indicated that the keratinases from both the wild-type and the mutant strain were relatively stable. Upon incubation at pH 7.5 for 12 h, the keratinases from the wild-type and the mutant strain retained 60% and 64% activity, respectively. After incubation at 40 °C for 12 h, however, the wild-type and the mutant strain keratinases showed 54% and 59% residual activity, respectively. In a recent study on keratinolytic enzymes, Liu et al. [65] reported the stability of recombinant keratinase from *Bacillus subtilis* at 10–50 °C and pH 7–11.5. Similarly, keratinase stability at pH 5–10 and temperature of 20–60 °C was also reported by Tork et al.[30] It could, therefore, be inferred from these results that the keratinase from the mutant strain was more stable than the one from the wild-type strain.

### Substrate specificity of the keratinases

Crude keratinases exhibited the most potent activity for feathers and this activity was assumed to be 100%. The activity of the wild-type and the mutant strain keratinases for human hair relative to that for feathers was 32.08% and 25.86%, while the activity for bovine hair was 41.98% and 36.12%, respectively. The enzyme, especially the keratinase from the mutant strain, was obviously more active towards feathers than towards bovine and human hair. The crude keratinase from *Pseudomonas stutzeri* K4 has been reported to show high substrate specificity for keratin and chicken feathers, whereas low specificity for collagen, casein and hair.[9] The characteristics of the keratinase in our study indicated that it could be adapted to a broad range of substrates, which could be applicable in keratinous waste treatment, medicine and cosmetic development.

### Effect of metals on keratinolytic activity

**Figure 4.**
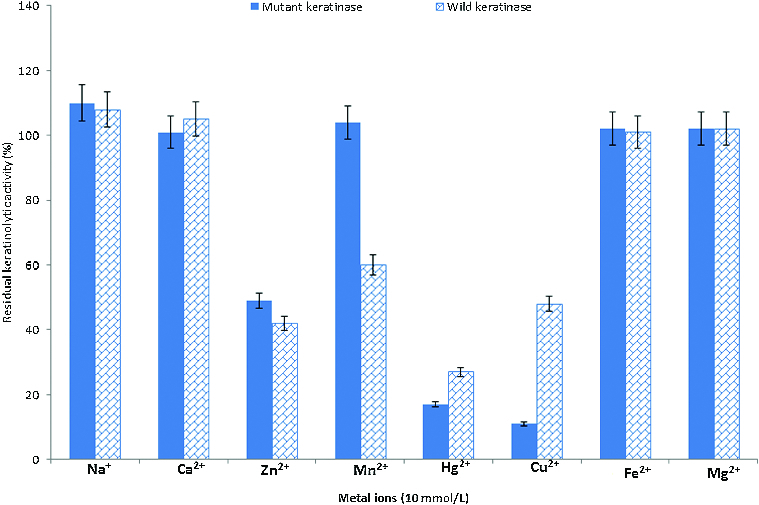
Effect of metal ions on the KA of *B. safensis* LAU 13 wild-type and mutant strain.

The results from our experiments on the effects of metals on the KA are as shown in Figure 4. The activity of extracellular crude keratinases from the wild-type and the mutant strain was stimulated in the presence of some metals, such as Na^+^, Ca^2+^, Mg^2+^ and Fe^2+^ with 100%–110% relative activity. On the other hand, the enzyme activity was inhibited in the presence of Hg^2+^, Cu^2+^ and Zn^2+^, with 10%–50% relative activity. In addition, while Mn^2+^ stimulated the activity of the enzyme from the mutant strain (104%), it inhibited the activity (60%) of the enzyme from the wild-type strain. Some reports have shown that keratinase activity can be stimulated in the presence of Na^+^, Ca^2+^ and Mg^2+^ and repressed by Hg^2+^, Cu^2+^ and Zn^2+^.[66,67]

### *In situ* degradation of whole chicken feathers

**Figure 5.**
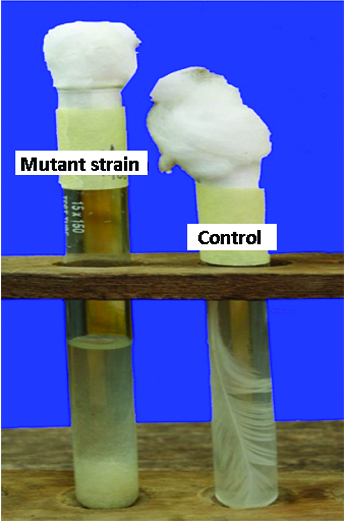
Complete degradation of a whole feather by *B. safensis* LAU 13.

The aerobic growth of the wild-type and the mutant strain on feathers as the primary source of carbon, nitrogen, energy and sulphur, resulted in complete degradation of the feathers after 9 and 6 days of incubation, respectively. The barbules and rachises were completely degraded by both strains to fine granulated forms and settled at the bottom of the test tubes (Figure 5) as previously observed in a similar study.[68] There are reports of complete or partial degradation of chicken feathers by bacteria in the range of 4–10 days.[7,9,69–71] It has also been reported that in many strains, complete degradation of feathers was not achieved, as the rachis was not fully degraded.[72] However, in our study, rachises were completely degraded by both the wild-type and the mutant strain, indicating that the two strains can completely degrade chicken feathers. These results were an indication that this bacterium could be useful in the biotechnological management of poultry feathers through efficient biodegradation.

### Dehairing of goat skin by the keratinases

**Figure 6.**
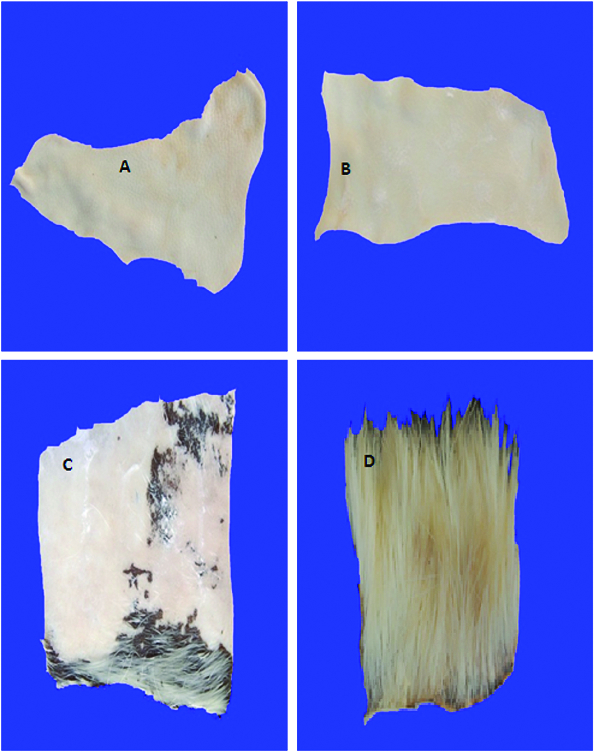
Complete dehairing of goat skin by crude keratinase. Note: Dehairing by wild-type strain after 16 h (A) and by mutant strain after 12 h (B); incomplete dehairing by sodium sulphide and lime after 20 h (C) and control (D).

The crude keratinases from the wild-type and the mutant strain completely dehaired goat hide without affecting the skin quality after 16 and 12 h of incubation, respectively (Figure 6). On the other hand, chemical dehairing lasted more than 20 h of incubation, without complete removal of the hair from the goat skin, as parts of the hair were still seen on the skin. Furthermore, the skin quality was partially damaged. The control skin that was incubated in distilled water, under identical conditions, showed no sign of hair removal. Leather processing industries generate a lot of toxic pollutants, such as sulphide and chromate, which are found to be detrimental to the environment.[73] Therefore, dehairing, using a microbial keratinase, is considered an easy alternative.[9] There are many reports of dehairing of goat/bovine skin, employing purified/semi purified keratinases.[9,27,74,75] Crude keratinase provides advantages, because it is cheap and easy for handling and industrial application. The crude keratinases of both strains might have accelerated the dehairing process, due to the synergistic action of different enzymes, which would have been removed during enzyme purification. In addition, the time required for complete enzymatic dehairing of goat skin, using the enzyme from the mutant strain, was shorter than that required by some others reported earlier.[9,54] This was an indication that keratinase from this strain could be a potential candidate for application in leather industry to avoid pollution problems associated with the use of chemicals in the industry.

### Destaining of blood-stained fabric

**Figure 7.**
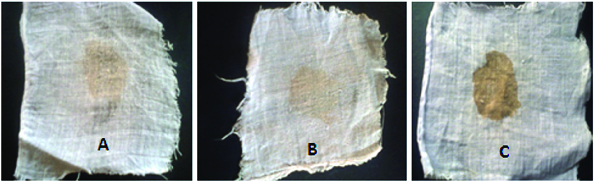
Destaining of blood-stained cloth by crude keratinases. Note: Destaining blood by the wild-type strain after 3 h of incubation (A), by the mutant strain after 2 h of incubation (B) vs. a control (C) incubated in water.

We observed remarkable removal of blood stains from white fabric after 3 and 2 h of incubation at room temperature (30 ± 2 °C), using keratinolytic enzymes from the wild-type and the mutant strain, at a minimum concentration of 57.4 and 89.2 U/mL, respectively (Figure 7). Kumar and Bhalla [25] reported the removal of egg yolk stains from fabric by protease (100 U/mL) after 2 h incubation at 40 °C. Similarly, Paul et al. [76] recently reported a remarkable removal of blood, egg yolk and chocolate stains safely from clothes by crude keratinase from *Paenibacillus woosongensis* TKB2. In our study, the keratinolytic enzyme obtained from the mutant strain destained faster than the enzyme from the wild-type strain. Also, stain removal by the enzyme from the mutant strain required a lower concentration than what was previously reported. This result further showed that keratinase is a promising tool for the removal of proteinaceous stains such as keratin, blood, egg yolk and other body secretions on fabrics. Therefore, the keratinolytic protease produced by this strain could be an appropriate detergent additive.

## Conclusions

This study led to the isolation of a novel keratinolytic strain of *Bacillus safensis* LAU 13 and the generation of its mutant variant through exposure to UV radiation. To the best of our knowledge, this work is the first reference to *Bacillus safensis* as a producer of keratinase with a remarkable potential in the biodegradation of feather wastes, destaining of blood-stained fabric and dehairing of animal skin. It was demonstrated that the mutant strain, in all cases, performed better than the wild-type strain, in respect of the utilization of the organism or the enzyme, as biocatalysts. It can, therefore, be concluded that a new vista has been opened in the biotechnological application of *Bacillus safensis* as a novel source of keratinases.
